# Uric Acid Is Elevated in Children With Obesity and Decreases After Weight Loss

**DOI:** 10.3389/fped.2021.814166

**Published:** 2022-01-04

**Authors:** Rasmus Møller Jørgensen, Bjarke Bøttger, Esben Thyssen Vestergaard, Britta Kremke, Rikke Frederiksen Bahnsen, Bent Windelborg Nielsen, Jens Meldgaard Bruun

**Affiliations:** ^1^Steno Diabetes Center Aarhus, Aarhus University Hospital, Aarhus, Denmark; ^2^Regionshospitalet Randers, Randers, Denmark; ^3^Department of Clinical Medicine, Faculty of Health, Aarhus University, Aarhus, Denmark; ^4^Danish National Center for Obesity, Aarhus, Denmark

**Keywords:** children, childhood obesity, uric acid, long-term follow-up, weight reduction, pediatrics

## Abstract

**Introduction:** Childhood obesity is an increasing condition associated with continuous obesity into adulthood and development of comorbidities. Adult studies show an association between serum uric acid (SUA) levels and body mass index (BMI). The aim of this retro perspective exploratory study was to investigate SUA in obese children and adolescents and the effects of a subsequent weight reduction.

**Materials and Methods:** One hundred and seventy-one children (age 4–18), with obesity (i.e. BMI-SDS of +2 or higher) were included in a multifactorial lifestyle intervention. The children participating were annually measured for anthropometrics, blood samples and DEXA-scans for up to 3 years. Eighty-nine children were included for follow-up analysis.

**Results:** After a follow-up of 20.7 ± 9.4 months a reduction in BMI-SDS of −0.34 ± 0.53 (*p* < 0.01) was observed. SUA was found to be positively associated with changes in BMI-SDS. SUA levels decreased in the 65 children who lost weight during the trial, conversely, SUA increased in the 23 children who gained weight during the trial (*p* < 0.01 between groups).

**Conclusion:** SUA was found to correlate with measures of obesity and for the first time, this intervention demonstrates a positive relationship between SUA and weight reduction in children with obesity.

## Introduction

During the last two decades, the prevalence of obesity and type 2 diabetes mellitus (T2D) has significantly increased, especially in developed countries (1). The prevalence of obesity amongst Danish children has shown a similar tendency (2). In 2018, the Danish Health Authorities reported that almost 20% of children between 7–18 years of age were considered to be overweight, of which, 3–4% were obese (2). Overweight in children can be defined as weight in kilograms divided by height in meters squared (body mass index (BMI) kg/m^2^) above the 85th percentile and obesity as a BMI above the 95th percentile (3).

In childhood, obesity is rarely associated with metabolic diseases such as T2D or cardiovascular disease (CVD) (1). However, in children with obesity, thorough clinical and biochemical examination can reveal prediabetes, dyslipidemia and elevated blood pressure - all of which can be characterized as part of the metabolic syndrome (MetS) (4–6). Onset of obesity in childhood is associated with increased risk of continuous obesity into adulthood and development of T2D in adulthood (7, 8). Obesity in childhood is also associated with an increased risk of developing other diseases in adulthood, including CVD, hypertension, sleep apnea, and certain cancers (9–11). Furthermore, children with obesity has been reported to have a significantly lower quality of life and a higher risk of developing depression (12, 13). These risks constitute the necessity for early diagnosis and treatment of overweight and obesity amongst children and adolescents. Several intervention studies show promising result regarding short term (less than five years) weight reduction among children with obesity (14, 15). However, further investigation is still needed to develop and evaluate the effectiveness of interventions targeting childhood obesity, in terms of preventing and alleviating complication later in life.

In a systematic review and meta-analysis Simmonds et al. suggest that various anthropometric measures including assessment of BMI in childhood is a poor marker in terms of predicting adult disease (16). This highlights that further research is needed to entangle and potentially determine which tool or marker is the best in childhood obesity to identify individuals at high risk. It is well established that abnormal changes in biomarkers such as dyslipidemia, hyperglycemia, and hyperinsulinemia all are key markers when diagnosing prediabetes, T2D or MetS in children with obesity (17, 18). In line with this, Wallace et al. have called for a better screening tool in terms of identifying children and adolescents at risk, preferably before development of prediabetes and T2D (18).

Uric acid is the catabolic metabolite of purine decomposition. The presence of uric acid in plasma is partly from endogenous turn-over of purines in our DNA material and partly from catabolism of purines from our diet. Elimination of plasma uric acid is solely through renal excretion. An association between increased levels of serum uric acid (SUA) in adults and development of T2D and MetS has been reported (19). A widely used definition of hyperuricemia is SUA > 7.0 mg/dL (0.42 mmol/L) and 5.7 mg/dL (0.34 mmol/L) for women (20, 21). The normal range of SUA for children and adolescents varies amongst countries and the reference values are generally lower than for adults (20). Large-scale studies describe sex- and age-specific variations in SUA amongst healthy children and adolescents (20).

Though reports indicate that hyperuricemia in adults is associated with e.g. T2D and obesity, it is also associated with multiple other diseases such as kidney disease, kidney stones, gout, and hypertension (22). Thus, SUA is not a specific marker of obesity or T2D. Nevertheless, elevated SUA may be interpreted as a risk marker for development of T2D and recent studies also suggest that uric acid may contribute as a target in the treatment of T2D (19, 23).

Clearly, an association has been described between uric acid, obesity, and metabolic disease in adults (19, 20, 24–26). A cross-sectional study of 144,856 adults found that individuals with overweight were more likely to develop hyperuricemia (25). In line with this, a meta-analysis of 12 cohort studies found a positive non-linear relationship between SUA and incident T2D (19) and a longitudinal study of 14,442 individuals demonstrated that SUA was a strong and independent predictor of MetS. In children with obesity, these potential relationships are still not fully elucidated and only a single study has reported an association between SUA and weight changes in a group of children with both normal weight, overweight and obesity (27). A longitudinal study found a positive relationship between body composition (i.e. BMI) and levels of SUA (28) in children with obesity. In 2020 Niu et al. reported a relationship between baseline SUA and weight reduction in 52 children with obesity during a 6-week weight loss camp (24) and a recently published analysis from the Bogalusa Heart Study reported a relationship between baseline SUA and changes in BMI in children and adolescents (29).

The hypothesis is, that SUA can be altered in children with obesity, if a sustainable weight loss is achieved. The aims of this study were therefore to investigate the relationship between uric acid in children with obesity, including the effects of a subsequent long-term weight reduction.

## Materials and Methods

### Study Design

This project is a retro perspective exploratory study of data from a community based lifestyle intervention for Danish children with obesity. Data from anthropometrics has previously been published (14).

### Definition of Overweight and Obesity

A standard deviation score for BMI (BMI-SDS) was used as measurement for overweight and obesity. BMI values were converted to BMI-SDS using a validated Danish reference (30).

### The Children's Obesity Clinic's Treatment Protocol

As described in detail in (14), the Children's Obesity Clinic's Treatment protocol is a family-centered lifestyle intervention treatment for children with obesity. At the first visit (baseline), each child received an individualized treatment plan. Anthropometry (height, weight and waist and hip circumference) was measured and used to calculate BMI and BMI-SDS. Body composition was assessed using dual energy x-ray absorptiometry (DEXA) scans and bio-impedance technique. Finally, blood samples were obtained (lipid profile, fasting glucose, HbA_1C_, urate, thyroid hormones, liver enzymes and kidney status). The participants were invited to an outpatient clinic for yearly visits to a maximum of 3 years: one at baseline, after 1, 2 and 3 years.

### Subjects

A total of 199 children and adolescents between 4 and 18 years of age (mean 10.8 ± SD 3.1 years) participated in the lifestyle intervention treatment (14). 24 participants were excluded from the calculations due to missing baseline SUA measurement and 4 participants were excluded as the interval between baseline anthropometrics and biomarkers exceeded 100 days. In this report, 171 children were included for baseline analysis, of whom, 89 participants had includable follow-up-measurements. The children had a BMI-SDS above + 2 SDs at baseline. They joined a treatment protocol containing lifestyle interventions and were annually measured with anthropometric values, DEXA scans and biomarkers. Participants with no blood samples at baseline were excluded from the calculations. If there were more than 100 days between the blood sample and the visit in outpatient clinic, respective measurements were excluded. The 89 included participants were divided into groups regarding change in BMI-SDS and in fat mass percentage obtained by DXA-scan (DEXA-FM). Children in Group A reduced BMI-SDS (*N* = 65) and children in Group B increased BMI-SDS (group B, *N* = 23) throughout the intervention. One participant had same BMI-SDS at the end of follow-up. This participant was excluded from analyses shown in **Tables 2**, **3**. Children in Group X reduced DEXA-FM (*N* = 51) during the intervention, while children in Group Z increased DEXA-FM (*N* = 34).

It was not possible to make a reasonable power calculation, because there were no prior reports in the literature investigating associations between weight change and SUA in an obese pediatric population.

### Biochemical Analyses

Blood was taken by puncturing v mediana cubitis. Blood samples were performed, transported to and analyzed by the local biochemical department. Immediately after being analyzed the blood was destroyed.

### Statistical Analyses

The data were collected and stored by using REDCap, an electronic data capture tool (31, 32). A single extraction of data was made. Statistical analyses were performed by using Excel (Microsoft Office Excel 2016). Continuous variables were converted to means with standard deviation (SD) and compared with student's *t*-test. A *p*-value of < 0.05 was considered statistically significant.

## Results

### Baseline Characteristics

One hundred seventy-one children had blood samples taken at baseline and were included in this trial, whereof 82 (48%) were girls. At baseline, the mean BMI-SDS was 3.13 kg/m^2^. Boys were significantly more overweight than girls (BMI-SDS 3.3 ± 0.7 vs. 2.9 ± 0.6, *p* < 0.01; Table 1). Anthropometric results have been reported earlier and as previously reported in the paper by Jørgensen et. al, the children in this trial had an overall reduction in BMI-SDS of 0.25 ± 0.56 kg/m^2^ (*p* < 0.01) and a beneficial change in body composition after participation in a multifactorial lifestyle intervention (14).

**Table 1:** Baseline characteristic (Anthropometric and biomarkers).

	**Boys**	**Girls**	***P*-value**
N	89 (52%)	82 (48%)	
Age, years	11.2 (3.4)	10.4 (2.8)	0.09
Height, cm	153.3 (20.0)	147.3 (14.6)	0.03
Weight, kg	67.1 (26.5)	59.8 (20.6)	0.05
BMI, kg/m^2^	27.2 (5.0)	26.7 (4.9)	0.51
BMI-SDS, kg/m^2^	3.3 (0.7)	2.9 (0.6)	<0.01
Waist circumferance, cm	94.2 (16.)	89.6 (13.8)	0.05
Hip circumferance, cm	97.0 (15.7)	95.1 (14.8)	0.40
bioimpedance fat, %	33.2 (6.3)	37.8 (5.2)	<0.01
bioimpedance muscle, %	63.6 (6.1)	59.1 (5.0)	<0.01
DEXA-FM, %	41.7 (4.7)	43.5 (4.7)	0.01
DEXA-FFM, %	55.6 (4.6)	53.8 (4.5)	<0.01
Cholesterol, mmol/L	4.2 (0.7)	4.3 (0.7)	0.63
HDL, mmol/L	1.3 (0.3)	1.3 (0.3)	0.49
LDL, mmol/L	2.6 (0.7)	2.7 (0.6)	0.46
Triglycerides, mmol/L	1.1 (0.8)	1.0 (0.5)	0.26
LDH, mmol/L	230.6 (40.5)	227.6 (49.7)	0.67
ALAT, IU/L	24.9 (13.8)	23.9 (16.4)	0.67
Bilirubin, μmol/L	7.4 (3.9)	7.1 (3.5)	0.61
Fasting glucose, mmol/L	5.2 (0.3)	5.0 (0.4)	0.03
HbA1c, mmol/L	5.6 (0.4)	5.6 (0.4)	0.50
TSH, 10^−3^ IU/L	2.6 (1.1)	2.5 (1.)	0.36
T3, pmol/L	6.3 (0.7)	6.4 (0.8)	0.51
Albumin, mg/L	41.2 (2.5)	41.1 (2.5)	0.86
Creatinine, μmol/L	48.6 (13.4)	44.2 (7.9)	<0.01
Carbamide, mmol/L	4.5 (1.1)	4.1 (1.0)	0.02
Uric acid, mmol/L	0.30 (0.09)	0.28 (0.06)	0.02

*The p-values represent differences between boys and girls. All data are reported as mean value with standard deviations (SD)*.

At baseline, boys had slightly higher fasting plasma glucose (5.2 ± 0.3 mmol/L vs. 5.0 ± 0.4 mmol/L, *p* = 0.03) and uric acid levels (0.30 ± 0.09 mmol/L vs. 0.28 ± 0.06 mmol/L, p = 0.02) as compared to girls. A similar significant difference between the two sex was observed in relation to levels of creatinine and carbamide (*p* < 0.01 and *p* = 0.02) (Table 1).

**Figure 1:**
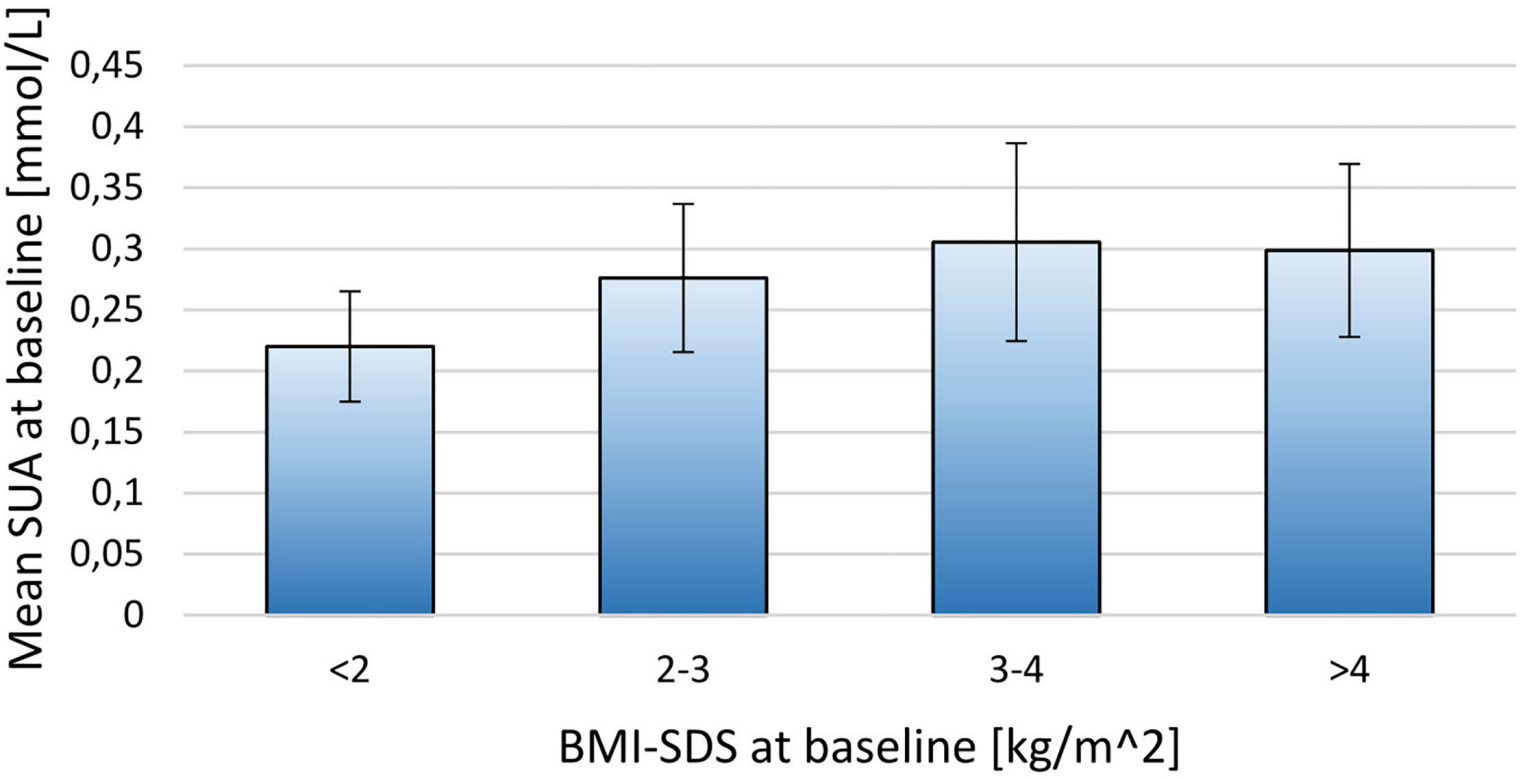
Stratifying mean SUA into four groups by BMI-SDS at baseline for the 171 included children. BMI-SDS <2 (*N* = 6), BMI-SDS 2-3 (*N* = 69), BMI-SDS 3-4 (*N* = 81), BMI-SDS>4 (*N* = 15). The thin lines represent SD.

**Figure 2:**
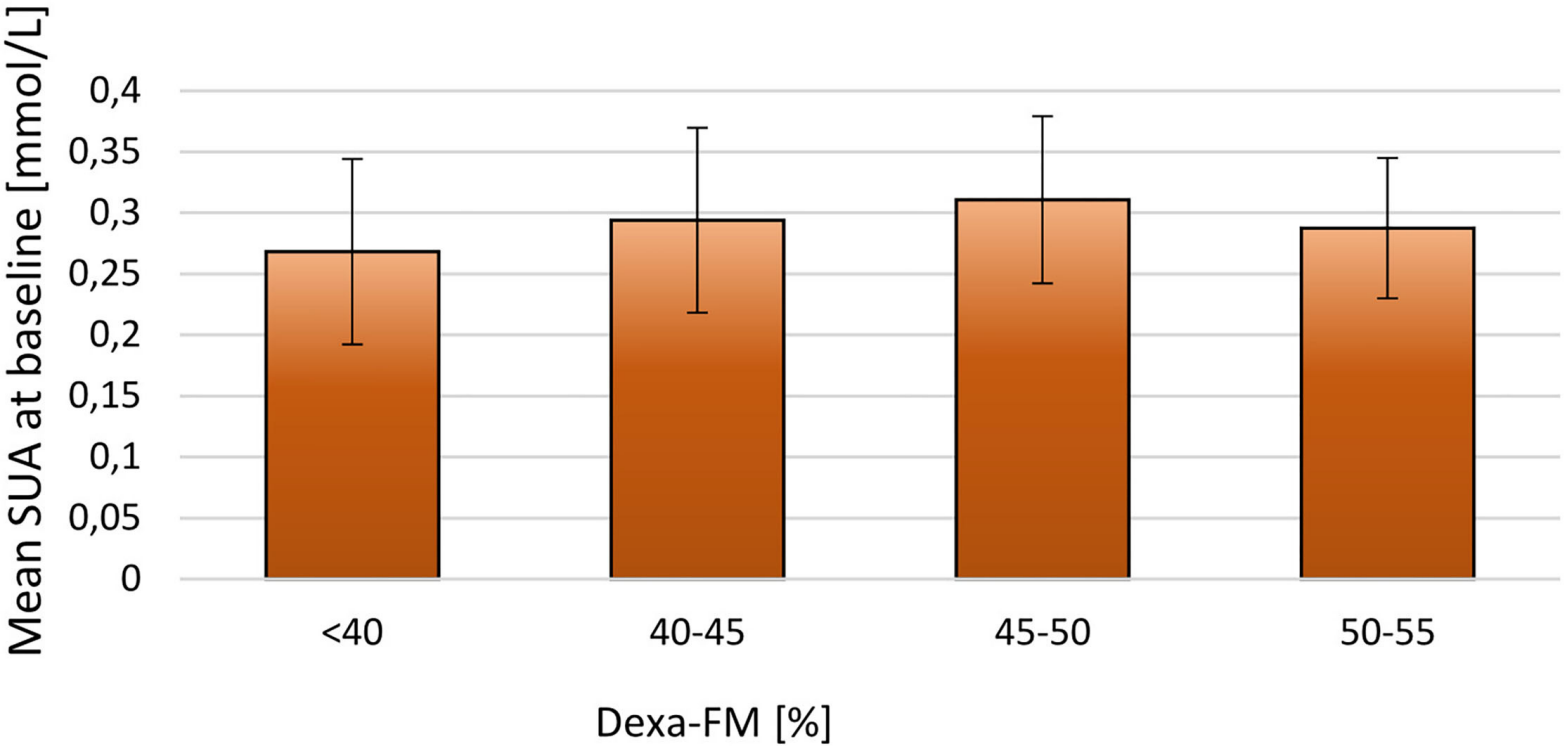
Stratifying mean SUA into four groups by DEXA-FM at baseline for the 171 included children. DEXA-FM <40 (*N* = 45), DEXA-FM 40-45 (*N* = 70), DEXA-FM 45-50 (*N* = 41), DEXA-FM 50-55 (*N* = 8). The thin lines represent SD.

When stratifying the population by BMI-SDS at baseline a positive relationship between SUA and adiposity was observed (*p* = 0.02; Figure 1). A similar, but non-significant tendency was observed when stratifying SUA by fat mass DEXA-FM (*p* = 0.06, Figure 2).

It was observed that children with BMI-SDS below median (3.1 kg/m^2^) had significantly lower SUA compared to the other half (0.28 ± 0.07 mmol/L vs. 0.30 ± 0.08 mmol/L, *p* = 0.02). Parallel differences were not observed in relation to cholesterol, carbamide, creatinine or HbA_1c_ levels (*p* = 0.26, *p* = 0.86 and *p* = 0.76, *p* = 0.33 respectively).

When dividing the children in those who had a sustained weight loss (group A) and those who had not (group B) at follow-up, baseline anthropometrics and body compositions were similar between the two groups (Table 2). In relation to biomarkers, only levels of bilirubin (mean 7.0 ± 3.3 vs. 5.6 ± 1.6; *p* = 0.01) were significantly different between the two groups at baseline.

**Table 2:** Baseline characteristic (anthropometrics) for the participants included in the follow-up analysis.

	**All**	**Group A**	**Group B**	***P*-value**
N	89	65 (73%)	23 (26%)	
Age, years	10.4 (2.9)	10.4 (2.6)	10.5 (3.9)	0.89
Height, cm	148.8 (17.1)	148.7 (14.8)	149.1 (23.1)	0.94
Weight, kg	60.8 (22.9)	59.5 (19.4)	64.1 (31.4)	0.51
BMI, kg/m^2^	26.4 (4.9)	26.1 (4.1)	26.9 (6.8)	0.61
BMI-SDS, kg/m^2^	3.1 (0.7)	3. (0.7)	3.1 (0.7)	0.63
Waist circumferance, cm	90.5 (14.9)	90.4 (13.5)	90.3 (18.9)	0.99
Hip circumferance, cm	94.5 (14.3)	94.1 (12.7)	95.3 (18.8)	0.78
bioimpedance fat, %	34.8 (6.1)	34.5 (5.9)	35.2 (6.7)	0.69
bioimpedance muscle, %	62.1 (5.8)	62.2 (5.7)	62.2 (6.2)	0.99
DEXA-FM, %	42 (5)	42 (4.9)	41.8 (5.2)	0.85
DEXA-FFM, %	55.2 (4.8)	55.2 (4.7)	55.4 (5)	0.85

*Group A consists of the children who reduced BMI-SDS and Group B consists of the children who increased BMI-SDS. The p-values represent differences between group A and group B. All data are reported as mean value with standard deviations (SD)*.

### Follow-Up

**Figure 3:**
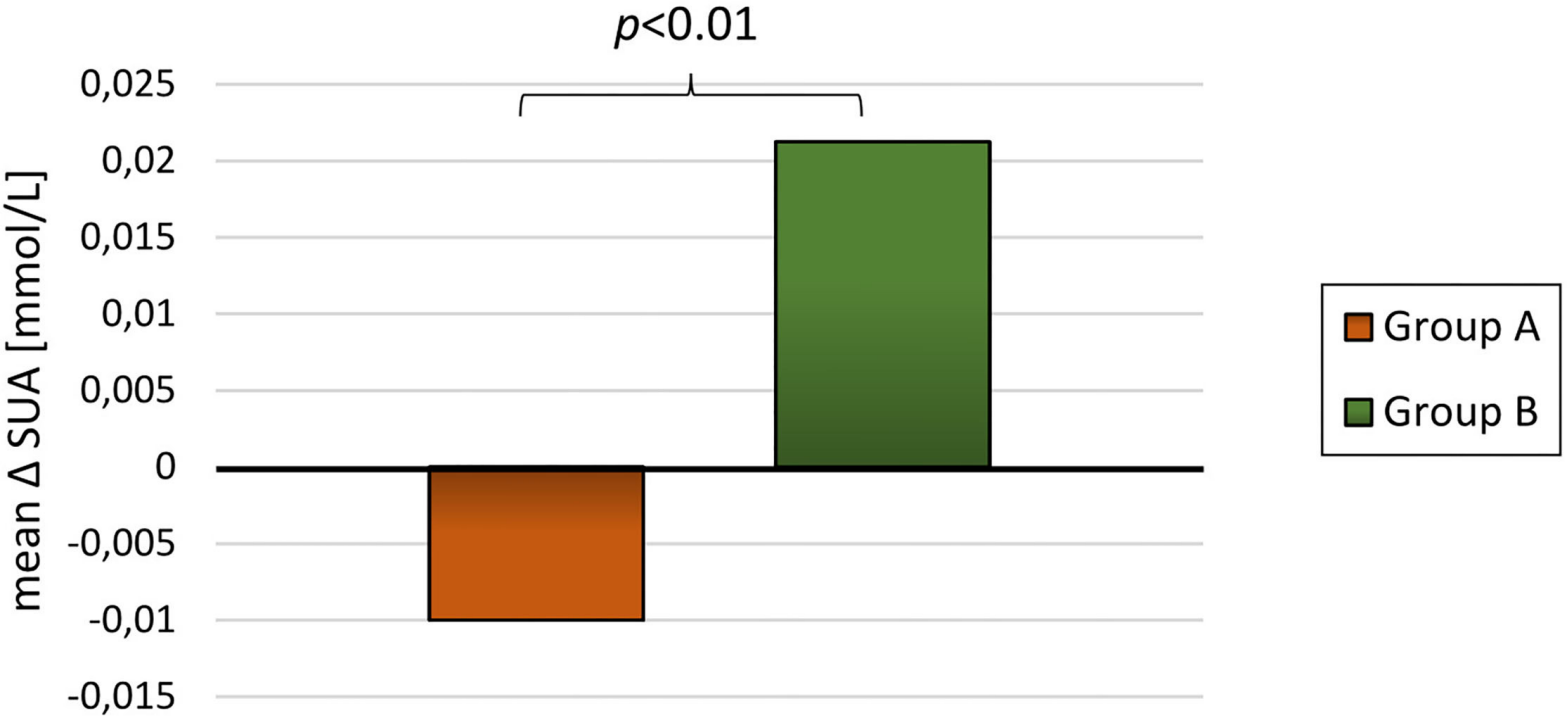
Mean delta SUA for group A (weight loss, *N* = 65) and group B (weight gain, *N* = 23).

**Figure 4:**
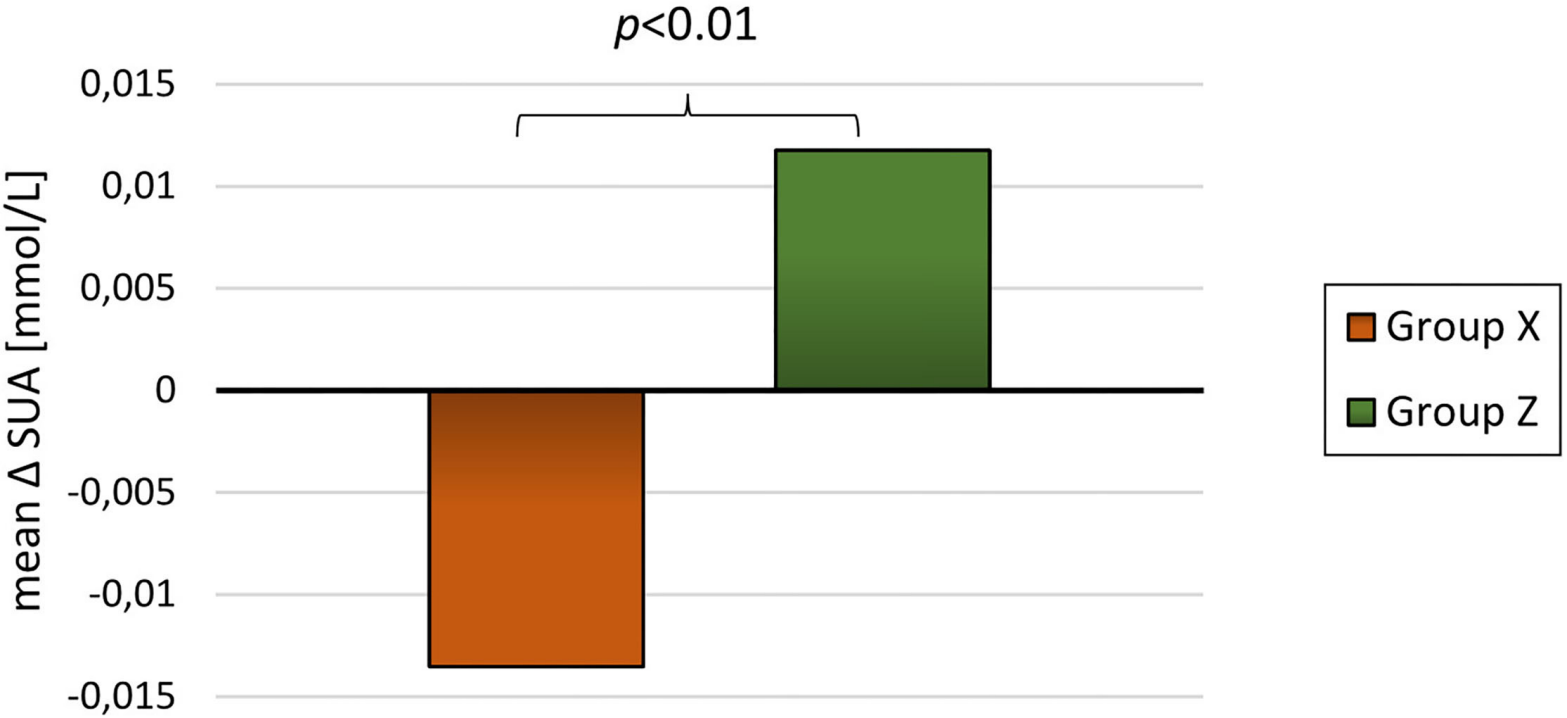
Mean delta SUA for group X (reduced DEXA-FM, *N* = 51) and group Z (increased DEXA-FM, *N* = 34).

For the 89 participants with blood samples at baseline and at least one subsequent blood sample the mean follow-up time was 20.7 ± 9.4 months. The participants had an overall reduction in BMI-SDS and DEXA-FM during this trial (BMI-SDS: −0.34 ± 0.53 *p* < 0.01, DEXA-FM: −2.17 ± 5.84 *p* < 0.01). When comparing delta values between group A and group B, it was observed that delta SUA was significantly different (*p* < 0.01, Table 3). SUA was observed to be reduced in group A and increased in group B (−0.01 ± 0.05 mmol/L vs. 0.02 ± 0.03 mmol/L; *p* < 0.01, Figure 3). Changes in triglyceride (*p* = 0.01) and HD-cholesterol (*p* < 0.01) were also significant different between the two groups (Table 3). When dividing the population based on DEXA-FM, a similar and significant difference in relation to SUA was found (Table 4, Figure 4). *Group X* consists of the children who reduced DEXA-FM and *Group Y* consists of the children who increased DEXA-FM.

**Table 3:** Follow-up delta-values (anthropometrics and biomarkers) for the participants included in the follow-up analysis.

	**All**	*P*-value^*^	**Group A**	**Group B**	*P*-value^**^
N	89		65 (73%)	23 (26%)	
BMI-SDS, kg/m^2^	−0.3 (0.5)	<0.01	−0.6 (0.4)	0.2 (0.3)	<0.01
DEXA–FM, %	−2.2 (5.9)	<0.01	−4.0 (5.7)	2.6 (2.8)	<0.01
Cholesterol, mmol/L	−0.1 (0.5)	0.1	−0.1 (0.5)	0.0 (0.6)	0.61
HDL, mmol/L	0.0 (0.2)	0.37	0.0 (0.2)	−0.1 (0.2)	<0.01
LDL, mmol/L	−0.2 (0.5)	<0.01	−0.2 (0.4)	0.0 (0.6)	0.09
Triglycerides, mmol/L	0.0 (0.6)	0.84	−0.1 (0.5)	0.3 (0.6)	0.01
LDH, mmol/L	−16.9 (33.0)	<0.01	−18.9 (34.3)	−11 (28.9)	0.31
ALAT, IU/L	0.9 (16.3)	0.61	−0.6 (7.8)	4.2 (29.3)	0.45
Bilirubin, μmol/L	1.8 (2.9)	<0.01	2.0 (3.2)	1.4 (1.9)	0.3
Fasting glucose, mmol/L	0.0 (0.4)	0.52	0.0 (0.4)	0.1 (0.4)	0.39
HbA1c, mmol/L	−0.1 (0.4)	0.04	−0.1 (0.4)	0.0 (0.4)	0.42
TSH, 10^−3^ IU/L	−0.1 (1.0)	0.2	−0.2 (1.1)	−0.1 (0.8)	0.72
T3, pmol/L	−0.2 (0.7)	0.03	−0.3 (0.7)	0.0 (0.7)	0.1
Albumin, g/L	−0.2 (2.6)	0.52	0.0 (2.7)	−0.9 (2.1)	0.11
Creatinine, μmol/L	3.6 (5.6)	<0.01	3.6 (5.4)	3.6 (6.2)	0.98
Carbamide, mmol/L	0.4 (1.3)	<0.01	0.2 (1.3)	0.7 (1.2)	0.11
Uric acid, mmol/L	0.0 (0.05)	0.98	−0.01 (0.05)	0.02 (0.03)	<0.01

*Group A consists of the children who reduced BMI-SDS and Group B consists of the children who increased BMI-SDS*.

*The p-values*

* *represent differences between baseline and last measurement*.

*The p-values*

** *represent differences between group A and B. All data are reported as mean value with standard deviations (SD)*.

**Table 4:** Follow-up delta-values (anthropometrics and biomarkers) for the participants included in the follow-up analysis.

	**Group X**	**Group Y**	***P*-value**
N	51 (60%)	34 (40%)	
BMI-SDS, kg/m^2^	−0.6 (0.5)	0.0 (0.3)	<0.01
DEXA-FM, %	−5.7 (4.9)	3.0 (2.1)	<0.01
Cholesterol, mmol/L	−0.1 (0.5)	0.0 (0.6)	0.26
HDL, mmol/L	0.1 (0.2)	−0.2 (0.2)	<0.01
LDL, mmol/L	−0.2 (0.5)	0.0 (0.5)	0.04
Triglycerides, mmol/L	−0.2 (0.4)	0.3 (0.6)	<0.01
LDH, mmol/L	−22.0 (35.8)	−7.3 (26.7)	0.04
ALAT, IU/L	−0.8 (8.6)	3.1 (24.3)	0.38
Bilirubin, μmol/L	1.9 (3.5)	1.6 (2.)	0.59
Fasting glucose, mmol/L	0.0 (0.4)	0.0 (0.4)	0.99
HbA1c, mmol/L	−0.2 (0.4)	0.0 (0.4)	0.02
TSH, 10^−3^ IU/L	−0.1 (1.1)	−0.3 (0.9)	0.18
T3, pmol/L	−0.3 (0.8)	−0.1 (0.7)	0.3
Albumin, g/L	−0.5 (2.6)	0.3 (2.6)	0.19
Creatinine, μmol/L	4.2 (5.9)	2.7 (5.1)	0.22
Carbamide, mmol/L	0.4 (1.4)	0.3 (1.1)	0.56
Uric acid, mmol/L	−0.01 (0.05)	0.01 (0.03)	0.01

*Group X consists of the children who reduced DEXA-FM and Group Y consists of the children who increased DEXA-FM. p-values represent differences between group X and Y. All data are reported as mean value with standard deviations (SD)*.

## Discussion

The present project is, to the best of our knowledge, the first to demonstrate a relationship between changes in SUA and changes in BMI-SDS during long-term follow-up in children with obesity. As depicted in Figure 2, children who obtained a weight reduction also had a significant reduction in SUA and in contrast, children with an overall weight increase had a significant increase in SUA. We found a dose-response between SUA and measures of adiposity (i.e. BMI-SDS) and thereby confirming previous reports in adults (25, 26, 33) and cross-sectional studies in children (28). Similar findings were reported by Viazzi et al., who demonstrated a positive association between weight development and SUA in children. However, Viazzi et al. had a shorter follow-up (≈ 1,5 years) and included children due to a risk of CVD and therefore only 48% of the participants were obese at baseline. These parameters make it difficult to directly compare it to our results (27). Using baseline levels of SUA, Niu et al. reported a positive relationship between SUA and weight reduction in children and adolescents with obesity (24). However, their study was limited to 53 participants submitted to a 6 weeks lifestyle intervention. Another recently published study has confirmed the relationship between baseline SUA and changes in BMI (29). The study examined the relationship between SUA and body composition by merging data from previous surveys into a longitudinal cohort study with a follow-up of 5–14 years. The authors reported an association between changes SUA and characteristics of MetS such as adiposity (i.e. BMI) and hypertension (29).

In relation to the opposite effects on SUA of weight reduction and weight gain, respectively, it is important to note, that reference values for SUA are age dependent with an increase in SUA reference values with increasing age in children and adolescents (34). Thus, a child with normal weight is expected to increase SUA levels during an equivalent follow-up period. Taking this into consideration, it is noteworthy to find a significant SUA reduction in the weight loss group.

The primary aim of this project was to investigate the relationship between SUA and weight loss. Baseline analysis, however, also demonstrates a relationship between SUA and BMI-SDS. Others have reported an association between SUA and body composition in children (20, 24, 27–29). The present calculations from this intervention supports these findings by describing an association between baseline SUA and BMI-SDS. In addition, SUA at baseline was significantly higher in the heaviest half of the population, when separated by median BMI-SDS.

However, even if SUA is related to body composition in children with obesity the mechanism by which SUA and BMI-SDS are related, is not fully understood. One of the theories suggests that the underlying disturbance in metabolism might be related to hyperinsulinemia, causing an elevated SUA in children with obesity (28). A thorough descriptive review suggest that hyperinsulinemia and/or insulin resistance may cause an impairment of the glycolytic pathway leading to accumulation of ribose-5-phosphate, which is a major substrate for uric acid production (35). In addition, hyperinsulinemia is known to modify renal excretion of uric acid in the kidneys, thus leading to elevated SUA (35).

Conversely, SUA may also promote or worsen insulin resistance. High SUA levels may inhibit nitric oxide bioavailability and since insulin requires nitric oxide to stimulate glucose uptake high SUA may induce insulin resistance (35, 36). Additionally, a substantial part of SUA arise from the diet (37) why the reduction in SUA found in group A may be a result of a lower dietary purine intake. Participants were encouraged to eat a healthy diet rich in vegetables and low on purine rich foods (e.g., red meat and bacon). A recent longitudinal study report that the relationship between SUA and BMI is bi-directional although the SUA-to-BMI path is stronger than the BMI-to-SUA path (38).

This intervention was not designed to investigate the underlying mechanisms of the relationship between SUA and changes in body composition. Future investigations on the relationship between changes in SUA and other biomarkers are therefore warranted.

Our study is the first to demonstrate that SUA is related to body composition and measures of adiposity (i.e. BMI-SDS) and displays beneficial changes in relation to weight reduction in children with obesity. This implies that SUA might be a useful marker both at baseline and in relation to weight loss. Previous studies have shown that obesity in children is associated with reduced quality of life in childhood and potential obesity-related complications later in life (9, 10, 12, 13). Several studies have called for better tools than BMI and BMI-SDS for identifying children and adolescents at risk (16, 18). Assessment of SUA may therefore contribute to the health assessment of children with obesity, however, further research should be made to validate the strength of SUA as a marker of development of adult comorbidities.

SUA may, however, not only be a marker, but an active component in development of metabolic diseases and obesity. Thus, it is hypothesized, that the increased disease risk associated with obesity is partially attributed to elevated circulating SUA (35). Assuming this, elevated SUA should be treated accordingly. The present study found that lifestyle intervention in children effectively lowered their SUA through weight reduction, supposedly, lowering their risk of disease.

Mortada et al. suggest that SUA-lowering medications could be applied to weight treatment (19) in terms of preventing T2D and in a study from 2005, Nakagawa et al. found that allopurinol could prevent fructose-induced hyperinsulinemia (39). Allopurinol lowers SUA by inhibiting xanthine oxidase - an enzyme responsible for degradation of purines into uric acid. It would be of great interest to investigate whether allopurinol could be used in children with obesity to support weight reduction and, especially, to reduce the risk of development of prediabetes, T2D and MetS.

The current projects is limited by the lack of a control group. Ideally, a randomized controlled trial should be performed to evaluate the effect of weight reduction on SUA levels. Additionally, the sample size of the intervention is rather small. Another weakness is that a large percentage of the children failed to complete the project due to the high complexity of the treatment and the interference with daily life.

An obvious strength of the present project is the long follow-up time and a very homogenous population and treatment. Also, a broad spectrum of biomarkers and anthropometric measurements enables extensive data analysis.

At conclusion the present study found SUA to be correlated with BMI-SDS in a dose-response related manner and demonstrated, for the first time, that changes in SUA is related to changes in bodyweight (i.e. weight reduction and weight gain) in children with obesity.

## Data Availability Statement

The raw data supporting the conclusions of this article will be made available by the authors, without undue reservation.

## Ethics Statement

Ethical review and approval was not required for the study on human participants in accordance with the local legislation and institutional requirements. Written informed consent for participation was not provided by the participants' legal guardians/next of kin because this intervention was designed and conducted as a community-based treatment for children with obesity. Danish legislation and the Ethical Committee does not require ethical approval and registration of such projects. Though the intervention was not a randomized trial, the CONSORT-concept was followed.

## Author Contributions

Healthcare workers in four municipalities were responsible for measuring anthropometrics between hospital visits. JB, RJ, and BB conceived the original idea for the study. RJ and RB were responsible for data collection. BB analyzed data and all authors had access to the data during the process. BB wrote first draft of the manuscript. All authors were involved in revision and final approval of the manuscript.

## Conflict of Interest

The authors declare that the research was conducted in the absence of any commercial or financial relationships that could be construed as a potential conflict of interest.

## Publisher's Note

All claims expressed in this article are solely those of the authors and do not necessarily represent those of their affiliated organizations, or those of the publisher, the editors and the reviewers. Any product that may be evaluated in this article, or claim that may be made by its manufacturer, is not guaranteed or endorsed by the publisher.

## Abbreviations

SUA: serum uric acid
BMI: body mass index
BMI-SDS: body mass index standard deviation score
CVD: cardiovascular disease
MetS: metabolic syndrome
T2D: type 2 diabetes mellitus
DEXA: dual energy x-ray absorptiometry
DEXA-FFM: dual energy x-ray absorptiometry scan fat-free mass
DEXA-FM: dual energy x-ray absorptiometry scan fat mass percentage.
